# The Harris Hawk (*Parabuteo unicinctus*) in Urban Areas of Argentina: Arrival in Mar Del Plata City and Green Area Use in Buenos Aires City

**DOI:** 10.3390/ani11041023

**Published:** 2021-04-05

**Authors:** Lucas M. Leveau

**Affiliations:** Departamento de Ecología, Genética y Evolución, Facultad de Ciencias Exactas y Naturales, Universidad de Buenos Aires–IEGEBA (CONICET–UBA), Ciudad Universitaria, Pab 2, Piso 4, Buenos Aires 1426, Argentina; lucasleveau@yahoo.com.ar

**Keywords:** birds of prey, invasion, Latin America, population, urban green area

## Abstract

**Simple Summary:**

The process of city colonization by raptors has been documented, if scarcely, in the Northern Hemisphere, whereas this kind of event has been seldom documented in the Southern Hemisphere. Additionally, raptor habitat use in urban areas has been scarcely studied in the Southern Hemisphere. The objectives of this study were: (1) to describe an event of Harris Hawk (*Parabuteo unicinctus*) arrival in Mar del Plata city, Argentina, and (2) to analyze its green area use in a recently colonized city, Buenos Aires. The Harris Hawk arrival was observed during 2019, mainly in periurban areas of Mar del Plata, and at least three pairs were breeding. In Buenos Aires, the occurrence of the Harris Hawk in green areas was related to the proximity to other large green spaces. The results obtained suggest the importance of green areas for raptor colonization in cities.

**Abstract:**

Urbanization has a negative impact on raptor species diversity and abundance. However, some species can adapt to urban areas, and the process of city colonization by raptors has been documented scarcely in the Northern Hemisphere. Information about city colonization by raptors in the Southern Hemisphere is null, and studies about habitat use by raptors are scarce. The objectives of this study were: (1) to describe an event of Harris Hawk (*Parabuteo unicinctus*) arrival in Mar del Plata city, Argentina, and (2) to analyze its green area use in a recently colonized city, Buenos Aires. Long-term data collected during 2002–2019, along an urbanization gradient of Mar del Plata, was used to describe the city arrival by the Harris Hawk. Surveys of green areas in Buenos Aires were used to model the Harris Hawk occurrence in relation to green area size and isolation to other green spaces. The Harris Hawk arrival was observed during 2019, mainly in periurban areas of Mar del Plata, and at least three pairs were breeding. In Buenos Aires, the occurrence of the Harris Hawk in green areas was related to the proximity to other large green areas. The results obtained suggest the importance of green areas for raptor colonization in cities.

## 1. Introduction

The continuous expansion of urban areas over natural and rural areas induces profound changes in raptor communities through habitat loss and fragmentation, decreasing raptor diversity and abundance [1,2,3,4,5,6,7]. Urban areas expose raptors to hazards, such as collisions with structures and vehicles, intoxication, diseases, and electrocution [8,9,10].

However, some raptors species may adapt to urban areas, especially those who have a generalist diet and use semi-open or forested habitats [11]. Moreover, the behavioral flexibility of certain raptor species is fundamental to survive in urban areas. For example, studies conducted on burrowing owls (*Athene cunicularia*) and Mississipi kites (*Ictinia mississippiensis*) have found that urban individuals exhibit higher tolerance to human proximity than rural individuals [12,13,14,15,16].

Due to the lack of long-term data, the establishment of raptor populations in cities has been scarcely documented [17,18,19]. The understanding of city colonization by raptors is important because it provides an opportunity to analyze invasion dynamics and promote efficient wildlife conservation in urban environments [18]. In general, the process of city colonization by raptors has been associated with an increase of prey abundance in urban areas and unfavorable conditions in rural areas, such as raptor persecution or a harsh climate [17,18]. City colonization events have been described in the Northern Hemisphere, whereas in the Southern Hemisphere, case studies are lacking.

Raptors in urban environments have shown to be associated positively to green area cover [9]. For example, the Cooper’s Hawk (*Accipiter cooperii*) in North America [19] and the Eurasian Sparrowhawk (*Accipiter nisus*) in Europe [20] have both been positively related to low-density residential areas and large urban parks, respectively. However, the breeding density of the Kestrel (*Falco tinnunculus*) in Rome (Italy) has been shown to be the highest in the urban center [21].

On the other hand, prey density in urban areas has also shown to be positively associated to raptor presence and abundance in urban areas [9]. For example, peregrine falcons in Warsaw (Poland) preyed mainly on the Feral Pigeon (*Columba livia*) [22], whereas tawny owls (*Strix aluco*) in Torun (Poland) preyed mainly on house sparrows (*Passer domesticus*) [23], and both prey species are common and resident in studied cities [22,23]. On the other hand, the Barn Owl (*Tyto alba*) and the Barred Owl (*Strix varia*) predominantly prey on rats (*Rattus* sp.) in urban areas [24,25,26].

The stable availability of prey and nesting places in urban areas has been associated with increased nesting success in raptors [27,28]. For example, nest boxes installed in urban Cape Town (South Africa) have been related to the population growth of the Peregrine Falcon (*Falco peregrinus*) [29].

The Harris Hawk (*Parabuteo unicinctus*) preys mostly on mammals and, to a lesser extent, birds [30]. However, the Harris Hawk can have an opportunistic diet [30], feeding on locally abundant prey, such as frogs [31]. On the other hand, the Harris Hawk inhabits semi-open habitats of Southern North America, Central America, and South America [30] and it has colonized several cities of central Argentina in recent decades. For example, in Buenos Aires, it was absent during the 1980s [32,33], whereas during the 2010s, the species was a common breeder in this city [34,35,36]. In La Plata, the Harris Hawk was absent during the 1990s [37,38], whereas since the 2010s, it has become a common raptor in the city (Carlos Leveau, Personal Communication). However, a detailed analysis of city colonization by the Harris Hawk is still lacking. Moreover, the habitat use of the Harris Hawk in the recently colonized cities has been scarcely studied.

The objectives of this study were: (1) to describe an event of city arrival by the Harris Hawk using long-term data collected along an urban gradient in Mar del Plata city and (2) to analyze the use of green areas by the Harris Hawk in a recently colonized city, Buenos Aires. The arrival of the Harris Hawk was expected to occur in periurban and suburban areas of the city, where nesting places and food resources are available. On the other hand, the habitat use of the Harris Hawk in Buenos Aires was expected to be related to large green areas.

## 2. Methods

### 2.1. Study Area

**Figure 1:**
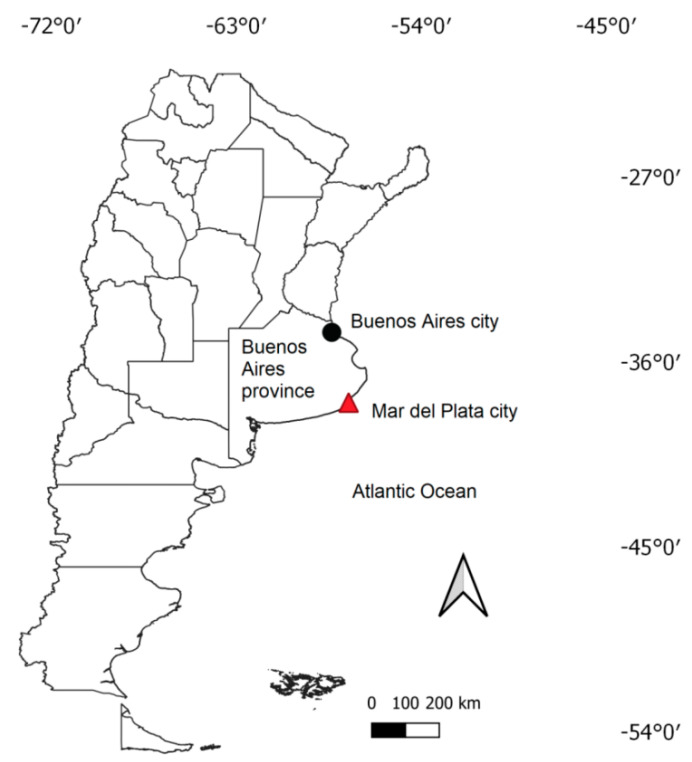
Location of Buenos Aires province, Buenos Aires city, and Mar del Plata city in Argentina.

The study was carried out in two cities of central Argentina (Figure 1): Mar del Plata (38°00′ S, 57°33′ W, 15 m asl; 593,337 inhabitants) and Buenos Aires (34°35′ S, 58°22′ W; 25 m asl; 3,075,646 inhabitants). Buenos Aires is located in the north-eastern area of Buenos Aires province, whereas Mar del Plata is located on the south-east coast of Buenos Aires province. The dominant surrounding matrix is composed of cropland and grassland used for cattle grazing. The climate in both cities is temperate, with an annual mean temperature between 14.0 and 17.9 °C and an annual precipitation of 923.6 and 1236.3 mm (Servicio Meteorológico Nacional, period 1961–1990).

### 2.2. Long-Term Survey in Mar del Plata

Bird surveys were carried out during the first four hours after dawn in December 2002–2005, 2009–2012, and 2018–2019. December coincides with the hawk breeding season in the Southern Hemisphere. One visit during each year was carried out in six transects; one located in the urban center, three in suburban areas, and two in periurban areas (Table 1). Transects were separated by at least 1.5 km from each other, and raptors were surveyed at both sides of the transect with an unlimited distance. The urban center was dominated by high buildings and commercial areas, the suburban areas had houses with yards, and the periurban areas were on the city fringe and composed of houses with yards and non-asphalted streets (see [39]).

### 2.3. Green Area Use in Buenos Aires 

A total of 23 green areas were visited, of which three were cemeteries and the rest parks. Unlimited radius point counts were carried out during October–December. For green areas of less than 4 ha, one point count was located, whereas the number of point counts for larger green areas increased according to their increasing areas. In 2019, while conducting a survey for a project about bird communities in cemeteries, three parks and three cemeteries were surveyed twice, giving 10 min for each one. In 2020, while conducting survey for a different project, the rest of the parks were surveyed three times, giving 5 min for each one. In both years, the bird surveys were performed by the same person (L.M.L.). Urban green areas were separated by at least 200 m from each other. Hawks were only counted when they were observed either perching or searching for prey within sampling areas.

Three environmental variables were measured: (1) area covered by the park or cemetery (mean = 10.96 ha, range = 0.70–79.81 ha); (2) minimum distance to green areas between 1 and 5 ha, which was called Dist1 (mean = 409.93 m, range = 6.36–1160 m); and (3) the minimum distance between green areas of more than 5 ha (mean = 748.10 m, range = 6.36–1850 m), which was called Dist2. These variables were measured by using Google Earth Pro. In addition, due to total survey length varied between green areas, this variable was incorporated in the models to control for survey effort.

### 2.4. Statistical Analysis

The relationship between Harris Hawk occurrence (presence/absence) in green areas and the environmental variables of Buenos Aires was analyzed with generalized linear models (GLMs), with a binomial distribution of errors in R [40]. The best model was obtained by running the model with all the environmental variables and gradually removing the non-significant variables until the final model was obtained (*p* > 0.05). The final model was compared with the null model through a likelihood ratio test (LRT) using the ANOVA function in R. There was no significant correlation between the continuous environmental variables (r < 0.70). The pseudo-rsquare of the final model was obtained using the function rsq of the rsq package [41]. The final model was plotted with the visreg package [42]. 

## 3. Results

In Mar del Plata city, the Harris Hawk started to appear in 2018 and the records increased notably in 2019 (Figure S1). Most of the records were made in periurban areas although some individuals were also observed in suburban areas. A total of nine juveniles and four adults were recorded. Nesting activity in the study area was evidenced by begging calls of juveniles and the nearby presence of adults. Due to the presence of begging calls by juveniles and adults nearby that were recorded in three transects, it is possible that at least three pairs were breeding during 2019. Moreover, an adult was observed carrying food to a juvenile. At the base of two trees were adults and juveniles were perched, remains of a Picazuro Pigeon (*Patagioenas picazuro*) were found.

**Figure 2:**
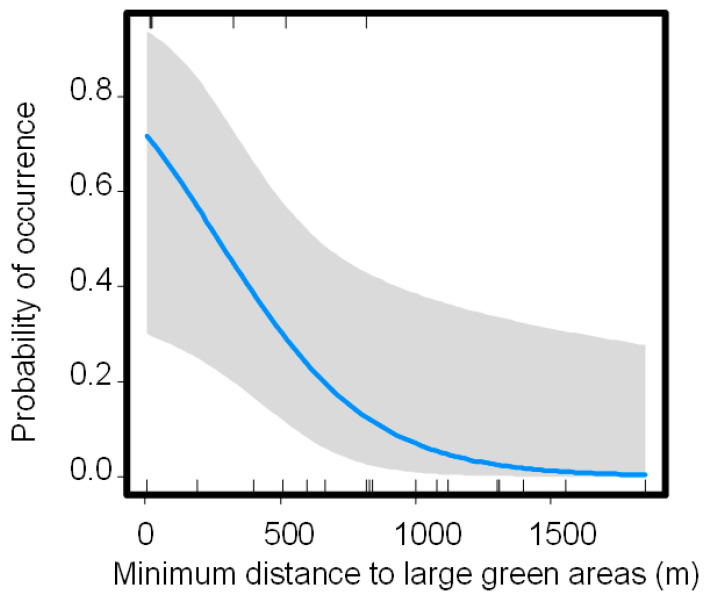
Harris hawk probability of occurrence in urban green areas in relation to the minimum distance to green areas of more than 5 ha (Dist2) in Buenos Aires city, Argentina. The vertical lines are distances between sampling sites with a presence (above X-axis) or absence (below X-axis). The blue line represents the fitted curve, and the grey area represents the confidence intervals at 95%.

In Buenos Aires city, the best model included Dist2 as the best variable related to Harris Hawk occurrence in green areas (pseudo r^2^ = 0.32; LRT = 7.93, *p* = 0.005; Table 2; Figure 2). Therefore, Harris Hawk occurrence was positively associated with green areas connected to large green areas (Figure 2).

## 4. Discussion

The results obtained showed that the Harris Hawk recently colonized Mar del Plata city, occupying periurban and suburban areas, with several vegetation strata yards. Moreover, the analysis of habitat use in Buenos Aires city showed that a network of large green areas was associated with Harris Hawk occurrence.

Begging calls by young individuals and the observation of an adult carrying food to young are strong evidences of breeding in the suburban and periurban areas in Mar del Plata city. These two facts support the process of city colonization by the Harris Hawk. The arrival of the Harris Hawk in Mar del Plata could be associated with at least three factors. Firstly, the Harris Hawk has been expanding its distribution in Buenos Aires province. For example, according to Narosky and Di Giacomo [43], the species was absent in the interior and the southeast coast of Buenos Aires province until 1993, whereas, according to eBird (accessed on 16 December 2020), the species has been present in several sites of the province interior and southeast coast in the 2010s. Therefore, during this distributional change, the Harris Hawk has colonized sites within urban areas. Secondly, the expansion and urban arrival can be related to an increase in potential prey, such as the Picazuro Pigeon, the Spot-winged Pigeon (*Patagioenas maculosa*), and the Eared Dove (*Zenaida auriculata*) [31,36]. Eared doves are abundant in urban areas of Buenos Aires province [44], and, according to personal unpublished data, their abundance has increased between 2004 and 2016 in Mar del Plata and Buenos Aires city. This increase in potential prey favoring urban raptor colonization has also been suggested in the case of Northern goshawks (*Accipiter gentilis*) in Hamburg, Germany [18]. Moreover, the Harris Hawk can use novel foraging behaviors to hunt locally abundant prey, such as frogs [31,45,46]. Thirdly, the presence of green areas with high trees in suburban and periurban areas constitutes potential nest substrates for the Harris Hawk [36].

Harris Hawk observations in green areas of Buenos Aires were positively related to the connectivity of other large green areas. This pattern indicates that the Harris Hawk probably needs several green areas to forage in the landscape. On the other hand, unlike other studies performed with raptors [20,47], the green area size was not an important factor explaining the Harris Hawk occurrence. Other environmental variables that could be affecting Harris Hawk occurrence in green areas, such as habitat structure, prey abundance, pedestrian traffic, and noise [20,48], have not been considered in this study. Therefore, results obtained here must be taken with caution. Moreover, the inclusion of more green areas is needed in future studies to get more insights related to habitat use of the Harris Hawk in Buenos Aires city.

## 5. Conclusions

The Harris Hawk is expanding its distribution in central Argentina, colonizing urban areas. In Mar del Plata, the Harris Hawk occupied periurban and suburban areas, both of which are composed by green areas for nesting and feeding. In Buenos Aires, the analysis of green area use by the Harris Hawk showed the importance of connected green areas in the city.

## Figures and Tables

**Table 1:** Transects characteristics used for the long-term survey of Harris hawks in Mar del Plata city, Argentina. Percentage mean cover of trees, lawn, and impervious surfaces were obtained from 25 m radius points (see Leveau and Leveau [39]).

Transect Name	Habitat	Length (km)	Tree Cover (%)	Lawn Cover (%)	Impervious Cover (%)
Downtown	Urban	3.00	5.77	0.03	94.00
Los Troncos	Suburban	2.08	22.82	9.38	68.55
Pinos de Anchorena	Suburban	1.41	24.50	20.53	54.80
Parque Luro	Suburban	1.36	22.00	15.50	65.50
Grosellar	Periurban	2.95	32.79	22.11	15.39
Bosque Peralta Ramos	Periurban	2.00	34.49	24.54	19.94

**Table 2:** The best generalized linear model (GLM), showing the relationship between Harris Hawk occurrence and the minimum distance to large green areas (Dist2) in Buenos Aires city, Argentina.

	Estimate	Std. Error	Z Value	*p*
Intercept	0.957	0.917	1.044	0.296
Dist2	−0.004	0.002	−2.181	0.029

## Data Availability

Data is available upon request to the author.

## Supplementary Materials

The following are available online at https://www.mdpi.com/article/10.3390/ani11041023/s1, Figure S1: Records (indivicuals/km) of the Harris Hawk during the period 2002–2019 in urban, suburban and periurban areas in Mar del Plata city, Argentina.
