# Supplementary material for: The Harris Hawk (Parabuteo unicinctus) in Urban Areas of Argentina: Arrival in Mar Del Plata City and Green Area Use in Buenos Aires City
Source: Animals (Basel). 2021 Apr 5;11(4):1023. doi: 10.3390/ani11041023 (PMC8066816; doi:10.3390/ani11041023)
Supplement: Supplementary file 1 [file animals-11-01023-s001.pdf]

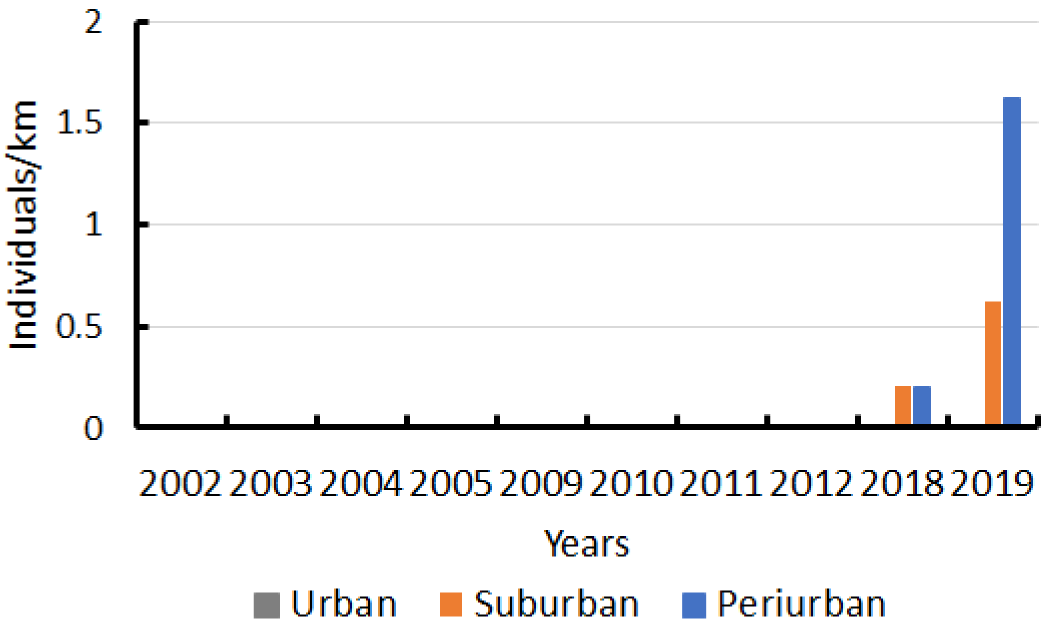

Figure S1. Records (individuals/km) of the Harris Hawk during the period 2002-2019 in urban, suburban and periurban areas in Mar del Plata city, Argentina.
